# Providing a Photovoltaic Performance Enhancement Relationship from Binary to Ternary Polymer Solar Cells via Machine Learning

**DOI:** 10.3390/polym16111496

**Published:** 2024-05-24

**Authors:** Jingyue Cao, Zheng Xu

**Affiliations:** 1 Key Laboratory of Luminescence and Optical Information, Beijing Jiaotong University, Ministry of Education, Beijing 100044, China; 17118456@bjtu.edu.cn; 2 Institute of Optoelectronics Technology, Beijing Jiaotong University, Beijing 100044, China

**Keywords:** polymer solar cells, machine learning, photovoltaic performance, molecular fingerprint, SHAP

## Abstract

Ternary polymer solar cells (PSCs) are currently the simplest and most efficient way to further improve the device performance in PSCs. To find high-performance organic photovoltaic materials, the established connection between the material structure and device performance before fabrication is of great significance. Herein, firstly, a database of the photovoltaic performance in 874 experimental PSCs reported in the literature is established, and three different fingerprint expressions of a molecular structure are explored as input features; the results show that long fingerprints of 2D atom pairs can contain more effective information and improve the accuracy of the models. Through supervised learning, five machine learning (ML) models were trained to build a mapping of the photovoltaic performance improvement relationship from binary to ternary PSCs. The GBDT model had the best predictive ability and generalization. Eighteen key structural features from a non-fullerene acceptor and the third components that affect the device’s PCE were screened based on this model, including a nitrile group with lone-pair electron, a halogen atom, an oxygen atom, etc. Interestingly, the structural features for the enhanced device’s PCE were essentially increased by the *J*_sc_ or FF. More importantly, the reliability of the ML model was further verified by preparing the highly efficient PSCs. Taking the PM6:BTP-eC9:PY-IT ternary PSC as an example, the PCE prediction (18.03%) by the model was in good agreement with the experimental results (17.78%), the relative prediction error was 1.41%, and the relative error between all experimental results and predicted results was less than 5%. These results indicate that ML is a useful tool for exploring the photovoltaic performance improvement of PSCs and accelerating the design and application with highly efficient non-fullerene materials.

## 1. Introduction

Polymer solar cells (PSCs) have seen their golden age in recent years with the development of non-fullerene acceptor (NFA) materials. Unlike fullerene-based materials with a low synthesis flexibility and high production cost, non-fullerene-based PSCs have the advantages of being low-cost, lightweight, and translucent, with mechanical flexibility and fast roll-to-roll printing [1,2,3,4,5,6]. At present, the ternary strategy of introducing the third component into binary PSCs is one of the research hotspots in this field. In other words, ternary PSCs contain an additional electron donor/acceptor material as the third component to break through the limitation of photon absorption by the host system, improve the active layer morphology, and adjust the energy level of the donor and acceptor to increase the power conversion efficiency (PCE) of the PSCs [7,8,9,10,11,12]. In addition, the amount of the third component may cause an increase in the energy and/or charge transfer and transport pathways, which can separately or simultaneously increase the device’s key photovoltaic parameter, such as the short-circuit current density (*J*_sc_), open-circuit voltage (*V*_oc_), and fill factor (FF) [13,14,15]. However, the effect of the type and the concentration of the third component on the performance of PSCs is not completely thoroughly understood. For a well-mixed binary donor–acceptor phase, when the third component causes the destruction of the phase structure, the free charge is easily trapped in the isolated domains, leading to severe amorphous recombination [16]. Moreover, the working mechanism of ternary PSCs is still controversial, which can be divided into the following four basic working principles: the energy transfer, charge transfer, alloy model, and parallel model [17,18,19,20]. All of these problems highlight the complex morphology of the active layer caused by the simple doping of third-component photovoltaic materials from binary to ternary PSCs. Therefore, a global, quantitative, and effective assessment of the complex relationship between the materials’ structural and performance enhancements from binary to ternary PSCs is badly needed.

In recent years, with the development of artificial intelligence, researchers have tried to use machine learning (ML) to build the structure–performance relationship described above. ML, as a technique for studying how to use computers to simulate human learning activities, aims to help researchers mine and understand the laws behind big data across a wide range of fields, such as the intersection of physics, chemistry, electronics, and materials science [21,22,23]. Nowadays, ML has been widely applied in the performance prediction of PSCs and the screening and design of new materials [24,25,26,27,28], and some work related to feature engineering and algorithm optimization to improve the prediction accuracy has been reported [29,30]. For example, Haibo Ma’s group used the gradient boosting regression tree (GBRT) model to conduct high-throughput screening on about 10,000 candidate materials, identifying important structural units and proposing 126 new material structures, with a predictive efficiency of more than 8% [24]. Akinori Saeki’s team utilized the extended connectivity fingerprints six (ECFP6) of the NFA’s chemical structure as input features to screen the polymers produced by units D and A, and matched them to highly effective donor materials blended with the ITIC acceptor; the final experimental PCE was 10.10% [25]. Di Huang et al. applied an ML model to quantify the important factors affecting the non-radiative voltage loss in non-fullerene PSCs, and then designed four new NFAs to minimize the voltage loss [26]. In addition, some researchers have introduced deep learning models to try to understand the physical properties of PSCs. Nahdia Majeed et al. developed artificial neural networks (ANNs) to quickly provide device parameters (including the carrier mobility, recombination rate, trap density, etc.), thus directly linking the manufacturing conditions with the device performance [31]. Youyong Li’s team built an automated framework for quickly achieving PCE prediction based on the molecular structure by combining deep learning and ensemble learning [32]. Moreover, our group determined the quantitative relationship between the molecular structure of thermally activated delayed fluorescent (TADF) emitters and the horizontal transition dipole moment ratio in host–guest films based on ML [33], and the intelligent screening of perovskite device interface passivation materials to minimize the voltage loss on p-i-n-type perovskite solar cells [34]. These studies all demonstrate the great potential of ML in the PSC field. However, existing work has mainly focused on predicting or analyzing the device performance in a single system, and few have provided insights into the data–mechanism hybrid drivers of performance improvement for high-performance ternary PSCs. Therefore, it is of great significance to construct the mapping relationship between binary and ternary PSCs with NFAs.

**Figure 1:**
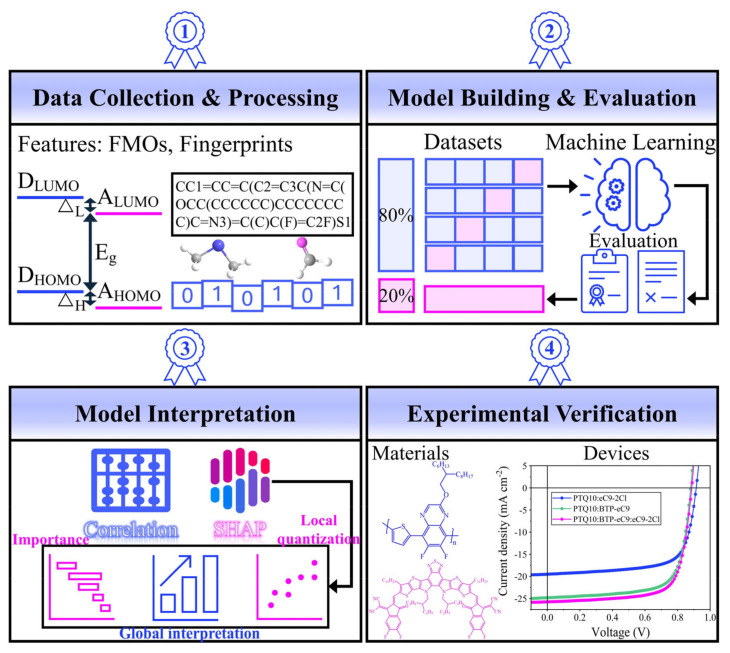
Flowchart of the machine learning mapping relationship established in this work.

In this work, as shown in Figure 1, we construct and interpret the mapping relationship of binary-to-ternary PSC photovoltaic performance improvements according to the following processes: data collection and processing, model building and evaluation, model interpretation and application, and experimental verification. Firstly, we investigated the significant influencing factors of the PCE in binary PSCs and tested the match between different types of molecular fingerprints and different algorithms in order to identify the most critical molecular structural units and the best model. The length of the fingerprints made a significant difference on the effective information contained by the molecules. In our models, the gradient boosting decision tree (GBDT) model had better adaptability to different molecular fingerprints than the other models on the whole. And the GBDT model was still stable in the ternary PSCs, and the key structural units selected based on the binary data were well transferred in the ternary data. In addition, we further screened the key structural features of the third-component materials, and it is interesting to note that the third component design guidance overlaps significantly with the design guidance from the binary acceptor materials. Finally, we fabricated four sets of efficient ternary and corresponding binary devices to verify the prediction model and the mapping relationship, and the model prediction results are in good agreement with the experimental results. Through this work, we provide a relationship between binary and ternary PSC photovoltaic performance improvements from the perspective of ML, which offers certain support for the quantitative research on PSCs.

## 2. Materials and Methods

**Materials:** PTQ10, PM6, BTP−eC9, eC9−2Cl, eC9−4F, Y6, L8−BO, PY−IT, and PDIN were purchased from Solarmer Materials Inc (Beijing, China). The PEDOT:PSS (Clevios P VP. AI 4083) was purchased from Xi’an Yuri Solar Co., Ltd. (Shaanxi, China). Chloroform (CF) and 1,8-diiodooctane (DIO) were commercially procured from J&K Scientific (Beijing, China) and Alfa Aesar (Shanghai, China), with purities of over 99.9% and 99.8%, respectively. All materials were purchased from suppliers and could be used without further processing. The PSCs were fabricated in the conventional structure of ITO/PEDOT:PSS/Active Layer/PDIN/Ag. For more a more detailed description about the solution preparation and device fabrication, see the Supplementary Materials. Then, the current–voltage (*J*-*V*) characteristics of the devices were measured by a Keithley 2400 Source Meter under simulated solar light (100 mW cm^−2^, AM 1.5G, Abet Solar Simulator Sun2000 from Zolix (Beijing, China)). All of the measurements were carried out at room temperature and in an atmospheric environment without any encapsulation.

**Methods:** This study focused on improving the photovoltaic performance of binary-to-ternary NFA-based polymer solar cells. The data set comprised the results of tests of the photovoltaic performance of 874 experimental PSCs collected from the literature. In order to obtain a more accurate mapping relationship and guidance for the design of NFAs, only the dual-acceptor (Donor/Acceptor1/Acceptor2, D/A1/A2) system was considered in the ternary devices. We used five types of supervised ML algorithms, including the decision tree (DT), random forest (RF), adaptive boosting (AdaBoost), eXtreme gradient boosting (XGBoost), and gradient boosting decision tree (GBDT). These are advanced algorithms that are described in detail in the Supplementary Materials. In addition, the data set was randomly divided by 8:2 into mutually independent training subsets and testing subsets. The training subset was used to train the model to establish the relationship between the input features and the target features. The testing subset was used to test the model to determine the prediction accuracy of the training model. And the coefficient of determination (R^2^), root mean square error (RMSE), mean absolute error (MAE), and mean absolute percentage error (MAPE) were often used to evaluate the performance of the models with regression problems. More importantly, we describe the electronic properties and chemical information from the materials in short ASCII strings, known as different molecular fingerprints (MFs), which are further elaborated below and in the Supplementary Materials.

## 3. Results and Discussion

**Figure 2:**
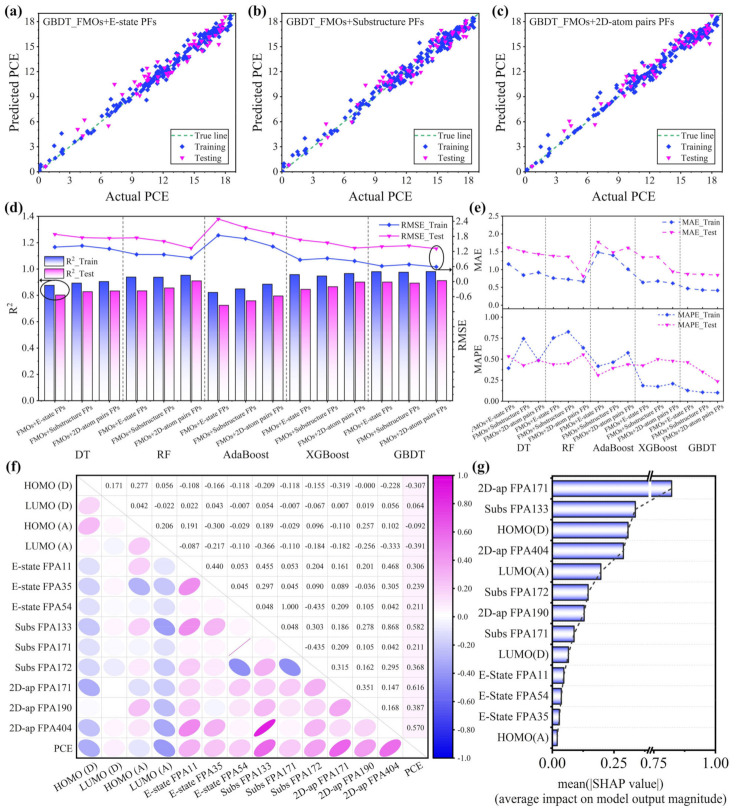
The fitting relationship between the PCE prediction results and experimental values reported in the literature based on the GBDT model: (**a**) FMO+E-state FPs, (**b**) FMO+Substructure FPs, and (**c**) FMO+2D-atom-pair FPs as input features. Performance indices of the models constructed by three different MFs and five ML algorithms on the training set and testing set: (**d**) R^2^ and RMSE, (**e**) MAE and MAPE. (**f**) The correlation matrix between the PCE and 13 key features; the value in the figure represents the linear correlation between the variables, namely, the *P*earson correlation coefficient. (**g**) Ranking the importance of the impact of the key input features on the PCE via the GBDT model. (**a**–**c**), the blue dots represent the training data, the magenta dots indicate the testing data, and the green dashed lines represent the visual references where the predicted values are equal to the experimental values.

In order to figure out the relationship between the molecular structure and device performance, it is necessary to convert the chemical structure into characters that can be recognized by a computer [35]. Different expressions of the same chemical structure contain very different information, and the ideal expression should cover almost all the features of the molecule. Therefore, we first compared the matching degree between three different MFs and the models constructed by five ML algorithms, and considered the influence of the fingerprint length on the model prediction performance in the three MFs. Among them, the E-state Fingerprint is a fingerprint descriptor that describes the electronic states of atoms and chemical groups in a molecule. It has 79 bits, which is short, but the similarity of the different fingerprints is relatively small [36]. The Substructure Fingerprint has 307 bits and encodes the presence or absence from a predefined set of chemical substructures in the molecule [37]. The 2D-atom-pair Fingerprint (780 Bit) encodes the existence of atomic pairs in the molecule and their topological distance in the molecule, which belong to relatively long fingerprints [38]. Figure S1 and Figure 2a–c show the results predicted in the five ML models (for the binary PSC data) using different types of fingerprints and frontier molecular orbitals (FMOs) as the input features, and Figure 2d,e summarize the corresponding performance indicators. Detailed data are recorded in Table 1. It is not difficult to find that the five ML models with the three different MFs all display good performance, which indicates that the MFs can effectively represent chemical information. In addition, overall, in each model, the 2D-atom-pair Fingerprint outperformed the other two fingerprints, possibly because longer fingerprints contain more chemical information. Among the five ML models, the GBDT model had the highest prediction accuracy for the PCE, followed by the XGBoost and RF, which may be attributed to its good generalization ability and its robustness on densely distributed data sets [39]. For the different fingerprints input into the GBDT model, the R^2^ of the E-state FPs, Substructure FPs, and 2D-atom-pair FPs obtained in the testing set were 0.901, 0.893, and 0.912, respectively, and the RMSEs were relatively low, at 1.395, 1.428, and 1.311; the MAE and MAPE also showed the same performance advantage. Since the performance difference in the GBDT model with the three FPs was not very large, we integrally applied the different expression information from the three molecular fingerprints to screen the key influencing factors on the performance of the binary PSCs.

To comprehensively screen the key influencing features and identify the differences between molecules, correlation analysis was used to investigate the multidimensional features. The *P*earson correlation matrix of the PCE with the donor and acceptor energy levels (four features) and the most critical nine features extracted from the three FPs for the binary PSCs are shown in Figure 2f. Table S1 gives a detailed description of these features. It is worth noting that the HOMO energy level of the donor (HOMO_(D)_) and the LUMO energy level of the acceptor (LUMO_(A)_) have obvious negative linear relationships with the PCE (*r*_HOMO(D)_ = −0.307, *r*_LUMO(A)_ = −0.391, respectively). That is, the deeper-lying HOMO_(D)_ and LUMO_(A)_ are conducive to improving the PCE of the device. Kuo Wang et al.’s study showed that the deeper-lying HOMO_(D)_ was conducive to reducing the energy range to increase the *V*_oc_ [28], while the deeper-lying LUMO_(A)_ generally contributed to the construction of narrow band-gap acceptors to broaden the NIR absorption, and thus increase the *J*_sc_ [40]. Usually, the effects of the LUMO_(D)_ and HOMO_(A)_ on the PCE seem to be ignored. In addition, the correlation between the E-state FPA11, 35, and 54 and the PCE is 0.306, 0.239, and 0.211, respectively, indicating that the introduction of =CH-, =O, and -Cl functional groups in the NFA design is beneficial to the improvement of the PCE. In a similar way, the Substructure FPs provide important functional groups of -C≡N, aryl bromide, and aryl fluoride. The nitrile group has a strong electron-withdrawing ability, which can narrow the band gap of the material. Halogens (F, Cl, and Br) have a different electronegativity, which can not only significantly affect the surface electrostatic potential of the molecules, but also affect the single-crystal packing behavior of the acceptors [41]. The introduction of oxygen atoms may create intramolecular conformational locking in some molecular structures, increasing the molecular planarity [42,43,44]. And the correlation between the 2D atom pairs FPA171, 190, and 409 and the PCE is 0.616, 0.387, and 0.570, respectively, which basically provides a constraint on the topological distance of the atoms in the molecules, such as the topological distance between the N-S, S-S, and N-O atomic pairs, which is ideally three, three, and six. This shows that the appropriate topological distance determines the strength of the interaction between each other, which further affects the electronic properties of the material itself. Further, in order to analyze the importance of the key features influencing the structure–performance mapping relationship, we identified the most critical features driving the prediction model based on the feature importance ranking of the best GBDT model. As shown in Figure 2g, the 2D atom pair FPA171 (the topological distance between N-S is three) is the top weight feature affecting the PCE. Both the central electron-deficient unit and the conjugate skeleton are supported by N-S in the adjacent position in Y-series acceptors. The ranking trend of the other features was basically consistent with the correlation analysis.

**Figure 3:**
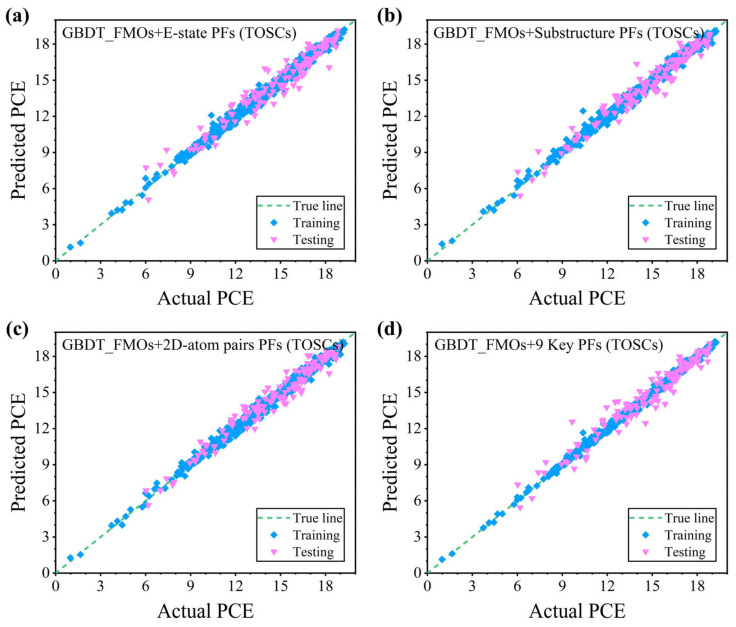
The fitting relationship between the PCE prediction results and experimental values reported in the literature based on the GBDT model in the ternary data: (**a**) FMO+E-state FPs, (**b**) FMO + Substructure FPs, (**c**) FMO + 2D-atom-pair FPs, and (**d**) FMOs + 9 Key FPs from the binary PSCs as input features. (the wathet dots represent the training data, the heliotrope dots represent the testing data, and the green dashed lines represent the visual references where the predicted values are equal to the experimental values).

To confirm the reliability of the constructed ML model, we established the mapping relationship between the ternary PSC material structure and the device performance based on the optimal algorithm, the GBDT. The binary devices were the basis and premise for fabricating the ternary devices, and how to build a data-driven bridge between them needs to be considered. Therefore, we recorded the photovoltaic parameters of the binary devices as the control parameters (C_*V*_oc_, C_*J*_sc_, C_FF, and C_PCE) and added the proportion of the third component (T%) together as the input feature. The prediction results of the GBDT model using three types of fingerprints, FMOs, and control parameters as input features are shown in Figure 3a–c, Figure S2 summarizes the corresponding performance indicators, and detailed data are recorded in Table 2. It is easy to find that, compared with the binary data, the prediction performance of the ternary PSCs in the GBDT model is further improved. And the E-state FPs, Substructure FPs, and 2D-atom-pair FPs in the testing set obtain R^2^s of 0.921, 0.928, and 0.934, respectively. Meanwhile, the RMSE, MAE, and MAPE are further reduced. This may be due to the fact that the control parameters from the binary devices provide a fairly good and efficient input to the training model. In addition, we applied the screened key features from binary PSCs into the ternary system for prediction, and a fairly good prediction result is shown in Figure 3d. The corresponding performance indicators, R^2^, RMSE, MAE, and MAPE, in the testing set are 0.908, 0.921, 0.695, and 0.053, respectively. This validates the key features and the important role of the NFAs in the ternary PSC performance.

For determining whether the addition of the third component in the ternary PSCs caused changes in the key features determining the PCE, *P*earson correlation analysis was applied, as shown in Figure S3. The negative correlation coefficient between the LUMO_(A)_ and PCE decreases from −0.391 to −0.072, while the correlation coefficient between the HOMO_(A)_ and PCE is significantly stronger than that of the binary PSCs. It seems that the HOMO_(T)_ has a certain positive effect on the HOMO_(A)_ (*r*_HOMO(T)_-_HOMO(A)_ = 0.220), that is to say, the addition of the third component changes the original energy level matching rules to some extent [45,46]. The negative correlation between the HOMO_(A)_ and PCE indicates that the deep-lying HOMO_(A)_ is beneficial to improving the PCE. This means that the third component breaks through the energy barrier and improves the charge transport by changing the smaller energy range between the main binary systems, which is usually reflected in the enhancement of the *V*_oc_ and FF. In addition, the third component ratio (T(%)) and PCE showed a significant negative correlation (*r*_T(%)_ = −0.352), indicating that the PCE decreased with the increasing content of the third component. This is because the addition of the third component makes the morphology of the active layer more complex, which depends on the miscibility phase of the donor–acceptor and third component [47,48]. When the third component content is 100%, it goes back to the binary PSCs. Interestingly, the correlation between the PCE of the ternary PSCs and C_*J*_sc_ and C_FF with the binary control parameters is quite high (*r*_C_*J*sc_ = 0.723, *r*_C_FF_ = 0.459), which indicates that the prerequisite for obtaining efficient ternary PSCs is that the binary devices must have a good photocurrent and miscible phase. And the addition of the third component will further enhance the *J*_sc_ through light absorption complementation. However, there is a very small positive correlation, with an *r*_C_*V*oc_ of 0.072, between the PCE of the ternary PSCs and the C_*V*_oc_. Compared with the contribution of the *J*_sc_ and FF, the role of the *V*_oc_ in improving the PCE of ternary devices seems insignificant. Although some works have reported that third-component materials can increase both the *V*_oc_ and *J*_sc_, most third component additions present a tradeoff between the *V*_oc_ and *J*_sc_ in the process of increasing the PCE. Based on the effective guidance of NFAs in binary PSCs, the same structural fragment screening was applied for the third-component materials. The correlation between the E-state FPT32, 38, and 51 and the PCE is 0.500, 0.237, and 0.128, respectively, indicating that the presence of -:N:, -F, and the aromatic sulfur functional group in the third component is conducive to the enhancement of the PCE. Similarly, Substructure FPs provide important functional groups such as aryl bromide and oxygen atoms, etc. In particular, when ketone structural fragments are present, the PCE is adversely affected. The correlation between the 2D atom pairs FPT06, 91, and 249 and the PCE is 0.237, 0.396, and 0.468, respectively, which suggests that the topological distance between the C-F, N-N, and N-S atomic pairs is preferably one, two, and four. Interestingly, we found that the key structural fragments of the third component showed mostly the same molecular fragments in the key structures as the binary system, in particular with the introduction of the halogen and oxygen atoms. Based on the principle of similar dissolution, it is understood that similar double-acceptor systems may have good miscibility and lead to a better morphology, which is reflected in the improvement of the FF [49].

**Figure 4:**
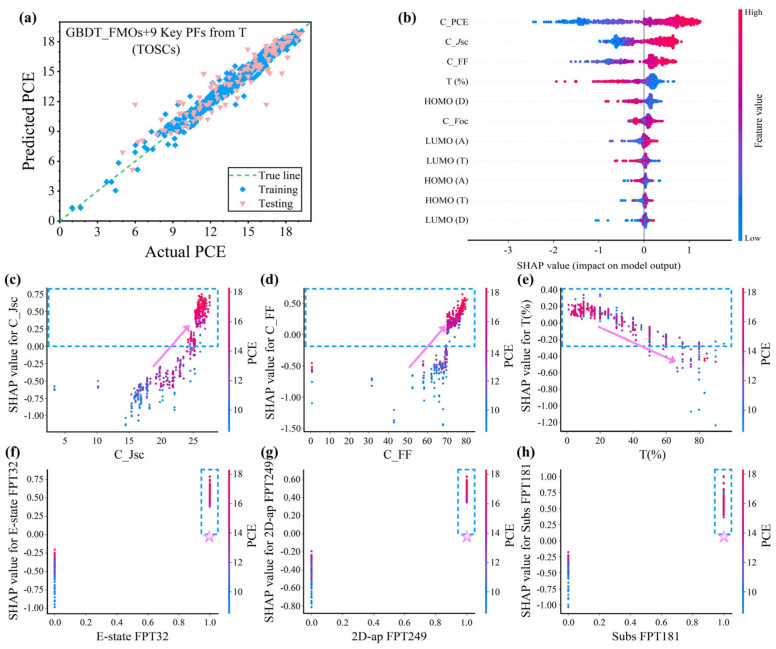
(**a**) The fitting relationship between the PCE prediction results in the ternary data and the experimental results reported in the literature based on the GBDT model with FMOs + 9 Key FPs from T as input features. (**b**) SHAP feature honeycomb map. The dependence of the (**c**) C_*J*_sc_, (**d**) C_FF, and (**e**) T(%) on the SHAP values varies with the PCE. (**f**–**h**) The dependence of the E-state FPT32, 2D-atom-pair FPT249, Substructure FPT181 and their SHAP values change with the PCE, respectively.

For verifying the effectiveness of the key structural features from the third component on the PCE prediction model, the GBDT model was again used to predict the PCE, and the results are shown in Figure 4a, Figure S4 summarizes the corresponding performance indicators, and detailed data are recorded in Table 2. The R^2^ for the testing set is 0.876. Because the third component is not the main acceptor in the ternary PSCs, its content in the ternary system is often far less than 50%, which explains the reason for the deterioration of the model performance. Then, the importance ranking of the key features affecting the performance in the ternary PSCs based on the GBDT model was analyzed, as shown in Figure S5a,b. The weight ranking trend of the key features was confirmed by correlation analysis. Herein, the SHAP interpreter was introduced to reflect the influence of the input features on the output features (PCE), which quantifies the positive or negative contribution of each input feature [50]. Figure 4b shows the global interpretation of the SHAP. The SHAP value corresponding to the distribution of the C_*J*_sc_ high-feature red points is greater than zero, which reveals that C_*J*_sc_ plays an active role in increasing the PCE, and the same goes for C_FF. Similarly, the SHAP value corresponding to the T(%) low-feature blue dot distribution is greater than zero, that is, the increase in T(%) has a negative effect on improving the PCE, which is consistent with the results of the *P*earson correlation analysis. All of these show that the PCE enhancement from the binary to ternary PSCs comes from the increased *J*_sc_ and FF. Further, we carried out a local interpretability analysis for the C_*J*_sc_, C_FF, and T(%). As shown in Figure 4c–e, the variation trend of the PCE with the C_*J*_sc_, C_FF, and T(%) is significant. And the C_*J*_sc_ is mainly concentrated from 25 to 27 mA cm^−2^, while the C_FF is mainly concentrated between 71 and 80%. However, T(%) shows an obvious decreasing trend with the increase in the content. In addition, we find that this trend seems to favor a low doping ratio of less than 20% for a high PCE. Since molecular fingerprinting uses “zero” and “one” to describe specific substructure information in molecules, it is not fully suitable for global interpretability analysis. Identifying the key structures from the third component from the local interpretation in Figure 4f–h tends to yield higher PCE values with high feature effects. In short, the SHAP makes the ML model no longer a so-called “black box”.

**Figure 5:**
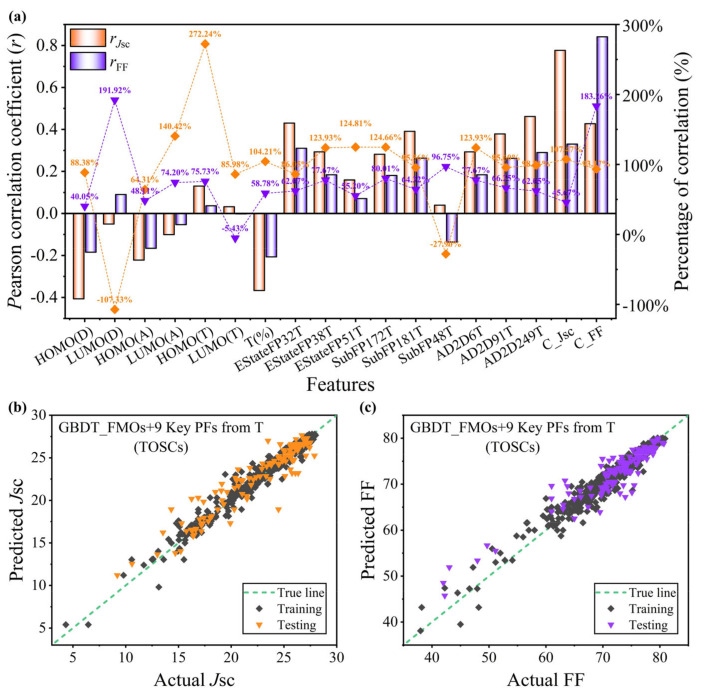
(**a**) The correlation coefficients between the *J*_sc_, FF, and key features of the ternary PSCs and the percentage of the correlation coefficients of the *J*_sc_ and FF divided by the correlation coefficient of the PCE, respectively. With FMOs+9 Key FPs from T as the input feature, the fitting relationship between the predicted results in the ternary data and the experimental values reported in the literature based on the GBDT model are as follows: (**b**) *J*_sc_; (**c**) FF.

To locate that the PCE improvement from the binary to ternary PSCs is dependent on the *J*_sc_ and FF, we quantified the impact of the important features screened from the ternary PSCs on the *J*_sc_ and FF, as shown in Figure 5a. We defined a correlation percentage to evaluate the specific impact of an input feature on the device performance improvement, which was equal to the correlation between the input feature and the *J*_sc_ and FF, respectively, divided by the correlation of the input feature with the PCE. It is not difficult to find that the HOMO_(T)_ accounts for 272.24% of the correlation in *r_J_*_sc_/*r*_PCE_, which indicates that the design of the HOMO_(T)_ is extremely important for the improvement of the *J*_sc_ of ternary PSCs, and the negative correlation ratio indicates that this feature is not achieved by improving the *J*_sc_ of the device PCE. Similarly, the LUMO_(D)_ exhibits a good correlation percentage (191.92%) in improving the FF of the ternary PSCs. However, the current design of the PSC donor and acceptor materials rarely take into account the LUMO level modification of the donor. Then, we predicted the *J*_sc_ and FF based on the key features from the ternary PSCs, and the results are shown in Figure 5b,c. The corresponding performance indicators are summarized in detail in Figure S6 and Table S3. The prediction performance of the model for the *J*_sc_ and FF is almost equivalent to that of the PCE, further determining the essential influence from the key features of the above screening on the performance improvement of the ternary PSCs.

**Figure 6:**
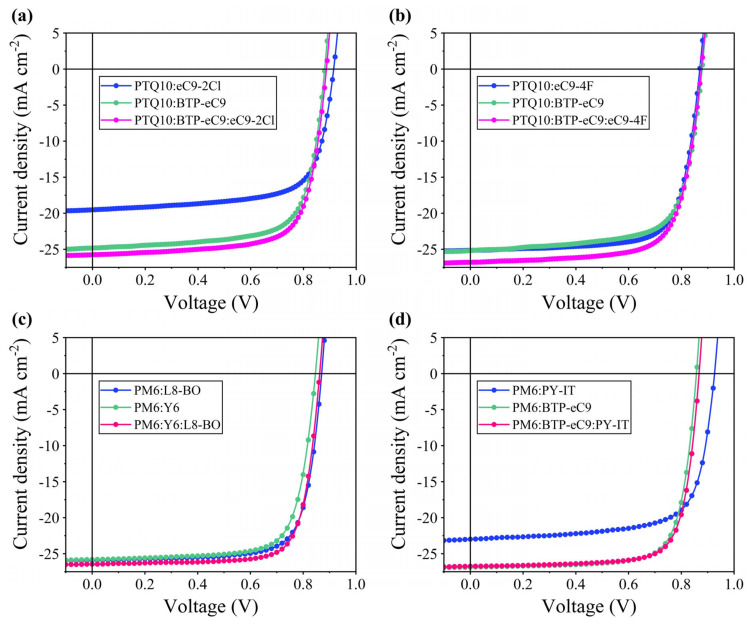
*J*-*V* characteristic curves of the binary and ternary PSCs with different active layers: (**a**,**b**) the active layers with PTQ10 as the donor; (**c**,**d**) the active layers in which PM6 is the donor.

In order to further verify the reliability of the established ML prediction model, the PTQ10 and PM6 popular materials were used as donors and matched to a variety of NFA materials to fabricate the binary and ternary PSCs, respectively. Under 100 mW cm^−2^ and AM 1.5G simulated sunlight irradiation, the device’s *J*-*V* characteristic curves and photovoltaic parameters are shown in Figure 6a–d and Table 3. Overall, the PCE of the ternary PSCs is improved compared with the corresponding binary devices, respectively, and almost all of them are from the improvements from the *J*_sc_ and FF, which is because the addition of the third component expands the light absorption in the main donor–acceptor system. For the two groups of PTQ10 ternary devices, the absorption of eC9-2Cl and eC9-4F has a certain blue-shift position compared with that of BTP-eC9, and the LUMO energy level of eC9-2Cl and eC9-4F rises, resulting in a wider band gap [51,52,53]. This is further evidence that complementary light absorption enhances the *J*_sc_ in ternary PSCs, and the widening of the band gap in the acceptors often leads to the blue shift of absorption, which is conducive to energy-level matching in the devices and the increase in the *V*_oc_, but, in this case, the increase in the PCE is often at the expense of the *V*_oc_. On the other hand, NFA end-group halogenation can effectively regulate the molecular energy levels, absorption, molecular crystallization, and mixed morphology. And the introduction of the strongly electronegative F atom can increase the molecular stacking, while the Cl atom has a higher polarization than the F atom to induce better molecular crystallinity, which is further reflected in the improvement in the devices’ FF [54,55,56,57]. This is consistent with the above statement that the introduction of F and/or Cl atoms in the NFAs or the third component can effectively enhance the PCE. For example, the FFs of the PTQ10:eC9-4F, PTQ10:eC9-2Cl, and PTQ10:BTP-eC9 devices are 73.58%, 70.25%, and 70.81%, respectively. The FF of the PTQ10:eC9-2Cl device is slightly lower than that of the PTQ10:BTP-eC9 device, perhaps because the strong crystallinity may lead to a larger phase separation, which requires more morphological characterization to reveal the complex morphological structure [51]. In both sets of PM6 ternary PSCs, the enhancement of the *J*_sc_ displays the same trend. Compared with Y6, L8-BO introduces branching alkyl side chains at the thiophene *β* position, which enhanced the miscibility and molecular packing behavior to a certain extent [57]. And the FFs of PM6:Y6, PM6:L8-BO, and PM6:Y6:L8-BO are 74.38%, 75.32%, and 76.90%, respectively. In addition, considering that polymer acceptors are also a very important branch of NFAs, for verifying the universality of the ML prediction model, PY-IT polymer acceptors were introduced to further prepare the corresponding devices. Unlike small-molecule acceptors, polymer acceptors tend to exhibit a high *V*_oc_ and the winding chain distribution pattern, so the PM6:PY-IT-based binary device has a relatively high *V*_oc_ of 0.926 V and a low FF of 71.46%. And the PM6:BTP-eC9:PY-IT ternary PSCs show a balance between the *V*_oc_ and *J*_sc_, and the FF is also relatively improved, which may be because a small amount of PY-IT forms the continuous fiber channel between it and the main system, which is helpful for improving the morphology of the active layer and carrier transport [58]. More importantly, the GBDT model’s PCE prediction results are in good agreement with the experimental test results, and the relatively small error (<5%) indicates that the prediction model can be extended to the prediction of the PSCs’ photovoltaic performance. Of course, the predicted results are still slightly different than the experimental results due to the inevitable differences in material batches, device structures, experimental conditions, test equipment, and other factors. In addition, it may result from the further optimization and improvement of the model generalization and robustness.

## 4. Conclusions

In summary, based on the database of the photovoltaic performance of the actual devices collected from the literature, a mapping model for the performance improvement of binary to ternary PSCs was constructed using various programming languages of the donor, acceptor, and third-component molecules, including FMOs and FPs, and the PCE of the corresponding PSCs was predicted. The results show that the relatively longer 2D-atom-pair Fingerprint (780 bit) provides the best fingerprint selection for the NFAs due to its efficient expression of chemical structure information. The GBDT algorithm was found to be more capable of handling complex and multidimensional input features, and it showed a better predictive ability and generalization in the binary data, ternary data, or with small amounts of information representation of key structural features. In addition, we not only found that the key structural fragments of the binary NFA material still have good applicability in the PCE prediction of the ternary data (the R^2^ was 0.908 in the testing set), but also determined that the introduction of halogen and/or O atoms into the third component structure enhanced the PCE, which overlapped with the key structural fragments from the binary system. More importantly, the relationship of the performance improvement from the binary to ternary PSCs is constructed from the point of view of ML; that is, the introduction of the third component often improved the PCE by increasing the device *J*_sc_ and FF, and provided some design guidance for NFAs in addition to the *J*_sc_ and FF, respectively. Finally, four sets of experiments were designed to prove the reliability of the ML model, and the prediction results of the ML model were basically consistent with all of the experimental results and have a relative error of less than 5%. Our research on the structure–device properties mapping of non-fullerene-based PSCs can accelerate the rational design and screening of the third component with ternary PSCs and accelerate the development of efficient PSCs.

## Figures and Tables

**Table 1:** The performance of the different algorithm models. The coefficient of determination (R^2^), root mean square error (RMSE), mean absolute error (MAE), and mean absolute percentage error (MAPE) were used to measure the above algorithm’s model performance (for binary data sets).

ML Models	Molecular Fingerprint Types	R^2^ Train/Test	RMSE Train/Test	MAE Train/Test	MAPE Train/Test
DT	E-state FPs	0.876/0.802	1.374/1.884	1.155/1.619	0.394/0.534
	Substructure FPs	0.893/0.828	1.426/1.753	0.843/1.504	0.745/0.426
	2D-atom-pair FPs	0.905/0.833	1.305/1.728	0.918/1.433	0.485/0.485
RF	E-state FPs	0.939/0.834	1.080/1.745	0.759/1.381	0.754/0.439
	Substructure FPs	0.938/0.856	1.075/1.604	0.725/1.360	0.825/0.451
	2D-atom-pair FPs	0.953/0.910	0.944/1.330	0.668/0.815	0.636/0.555
AdaBoost	E-state FPs	0.822/0.725	1.844/2.490	1.484/1.775	0.417/0.311
	Substructure FPs	0.849/0.759	1.708/2.154	1.398/1.481	0.464/0.394
	2D-atom-pair FPs	0.885/0.795	1.396/1.915	1.012/1.607	0.576/0.439
XGBoost	E-state FPs	0.958/0.846	0.866/1.661	0.637/1.342	0.187/0.425
	Substructure FPs	0.947/0.866	0.924/1.547	0.675/1.355	0.176/0.499
	2D-atom-pair FPs	0.966/0.901	0.821/1.334	0.613/0.943	0.210/0.479
GBDT	E-state FPs	0.980/0.901	0.619/1.395	0.467/0.874	0.129/0.463
	Substructure FPs	0.976/0.893	0.685/1.428	0.425/0.866	0.107/0.351
	2D-atom-pair FPs	0.981/0.912	0.587/1.311	0.412/0.845	0.102/0.237

**Table 2:** The performance of the GBDT model as measured by the R^2^, RMSE, MAE, and MAPE in the ternary data set.

ML Models	Molecular Fingerprint Types	R^2^ Train/Test	RMSE Train/Test	MAE Train/Test	MAPE Train/Test
GBDT	E-state FPs	0.991/0.921	0.307/0.854	0.239/0.636	0.019/0.049
	Substructure FPs	0.992/0.928	0.300/0.819	0.222/0.587	0.018/0.046
	2D-atom-pair FPs	0.996/0.934	0.218/0.781	0.168/0.552	0.013/0.043
	9 Key FPs from BPSCs	0.968/0.908	0.548/0.921	0.454/0.695	0.032/0.053
	9 Key FPs from T	0.935/0.876	0.778/1.190	0.561/0.796	0.044/0.069

**Table 3:** Experimental test results of the photovoltaic performance for the binary and ternary PSCs with different active layers and the PCE prediction results based on the GBDT model. The relative error (%) between the predicted PCE and experimental PCE, which is calculated as = [(Predicted PCE − Experimental PCE)/Experimental PCE] × 100%.

Systems	Devices	*V*_oc_ (V)	*J*_sc_ (mA cm^−2^)	FF (%)	PCE (%)	Predicted PCE (%)	Relative Error (%)
PTQ10	PTQ10:eC9-2Cl	0.915	19.53	70.25	12.55	13.12	4.54
	PTQ10:BTP-eC9	0.880	24.83	71.75	15.68	15.74	0.38
	PTQ10:BTP-eC9:eC9-2Cl	0.887	25.76	72.48	16.56	16.79	1.39
	PTQ10:eC9-4F	0.870	25.15	73.58	16.10	15.88	1.37
	PTQ10:BTP-eC9	0.878	25.20	70.81	15.66	16.12	2.94
	PTQ10:BTP-eC9:eC9-4F	0.876	26.80	72.06	16.92	17.14	1.30
PM6	PM6:L8-BO	0.871	25.91	75.32	16.99	17.31	1.88
	PM6:Y6	0.847	25.79	74.38	16.25	15.78	2.89
	PM6:Y6:L8-BO	0.863	26.46	76.90	17.57	17.86	1.65
	PM6:PY-IT	0.926	22.93	71.46	15.20	15.75	3.62
	PM6:BTP-eC9	0.858	26.87	76.11	17.55	17.23	1.82
	PM6:BTP-eC9:PY-IT	0.868	26.74	76.60	17.78	18.03	1.41

## Data Availability

Data are contained within the article.

## Supplementary Materials

The following supporting information can be downloaded at: https://www.mdpi.com/article/10.3390/polym16111496/s1, Figure S1: The fitting relationship between the PCE prediction results and experimental values reported in the literature using FMO + E-state FPs, FMO + Substructure FPs, and FMO + 2D-atom-pair FPs as the input features based on the five machine learning models, the DT, RF, AdaBoost, XGBoost, and GBDT, respectively; Figure S2: The performance evaluation indicators of the GBDT model based on the ternary data PCE prediction results on the training set and testing set: (a) R2, (b) RMSE, (c) MAE, and (d) MAPE with FMO + E-state FPs, FMO + Substructure FPs, FMO + 2D-atom-pair FPs, and FMOs + 9 Key FPs from the binary PSCs as input features, respectively; Figure S3: Correlation matrix of the PCE and (a) FMOs, T(%), and control parameter features; (b) 9 key features from the third component in ternary PSCs; Figure S4: The performance evaluation indicators of the GBDT model based on the ternary data PCE prediction results on the training set and testing set with FMOs + 9 Key FPs from T as the input feature; Figure S5: (a,b) Ranking the importance of the influence of key input features on the PCE of ternary PSCs in the GBDT model; Figure S6: The performance evaluation indicators of the GBDT model based on the ternary data: (a) Jsc and (b) FF prediction results on the training set and testing set with FMOs + 9 Key FPs from T as the input feature; Figure S7: The chemical structure of the donor, acceptor, and third-component materials used in this work; Table S1: The screened 9 key molecular fingerprint descriptors from the binary data; Table S2: The screened 9 key molecular fingerprint descriptors from the ternary data; Table S3: The R2, RMSE, MAE, and MAPE used to evaluate the GBDT model’s performance for Jsc and FF prediction on the ternary data set with FMOs + 9 Key FPs from T as the input feature.
