# Supplementary material for: Providing a Photovoltaic Performance Enhancement Relationship from Binary to Ternary Polymer Solar Cells via Machine Learning
Source: Polymers (Basel). 2024 May 24;16(11):1496. doi: 10.3390/polym16111496 (PMC11174796; doi:10.3390/polym16111496)
Supplement: Supplementary file 1 [file polymers-16-01496-s001.zip › polymers-3006254-supplementary.pdf]

Supplementary Information

Providing a photovoltaic performance enhancement relationship from binary to ternary polymer solar cells via machine learning

Jingyue Cao[1,2], Zheng Xu[1,2 *]
1, Key Laboratory of Luminescence and Optical Information, Beijing Jiaotong University, Ministry of Education, Beijing 100044, China.
2, Institute of Optoelectronics Technology, Beijing Jiaotong University, Beijing 100044, China.
*Corresponding author: zhengxu@bjtu.edu.cn

Experimental details:

Data set preparation:

The data set is derived directly from the literature on ternary polymer solar cells (PSCs) reported in recent years. In order to ensure the consistency of data collection standards and build high-quality data set, then only experimental results containing the photovoltaic parameters of the ternary PSCs and their corresponding binary PSCs are retained in the publication to reflect the current progress from ternary PSCs. The data set consists of 874 experimental results, including 263 sets of binary PSCs photovoltaic performance and 611 sets of ternary PSCs photovoltaic performance. And input features include the frontier molecular orbitals (FMOs) and molecular fingerprints generated using different SMILEs codes for the donor, acceptor, and third component materials. Most of the studies have used the ultraviolet photoemission spectroscopy (UPS) to measure the HOMO energy level of thin films, and inverse photoelectron spectroscopy (IPES) is used to measure the LUMO energy level of films. We use ChemDraw software to map the molecular structures of donor, acceptor and third component materials in the data set and generate their SMILEs codes, which are ultimately translated into the programming language representations of the molecules (different types of molecular fingerprints in this work) for rapid recognition

by machine learning (ML) models. In the data set, the *K*NN algorithm is used to fill in few missing features. In addition, the data set used for models learning is randomly divided into two parts. The training subset (80% of the total data set) is used to train the model to establish the relationship between input features and device performance. And the testing subsets (20% of the total data set) is used for model predictions to determine the prediction accuracy of the model.

Feature engineering:

In order to reduce dimensiona of the data set, the correlation analysis in the statistical model and feature importance analysis in the algorithm model are used. And the most critical structural features are selected from the various features describing the different molecular fingerprints of the donor, acceptor and third component materials for constructing the optimized photovoltaic performance prediction model with a set of low-dimensional feature descriptions as inputs to reduce the overfitting problem in the models. In addition, the screened feature importance is calculated using the gain type of the plot_importance function provided by lightgbm in Python. And the average of the training losses are applied to calculate the screened features importance. When each feature is used for tree segmentation, the average of the training losses is reduced. If the mean value of the training loss is significantly reduced when the input feature is used, this input feature can be considered to contribute significantly to predicting the ML model.

Model building:

In this work, learning models are built based on the Python language environment. And five type of algorithms (Decision tree (DT), Random forest (RF), Adaptive boosting (AdaBoost), eXtreme gradient boosting (XGBoost), and Gradient boosting decision tree (GBDT)) are used to evaluate the performance of model prediction. The DT algorithm is a commonly used ML algorithm, which makes decision prediction based on the tree structure, that is, divide the data gradually according to the features until the predetermined termination condition is reached. DT is also a conditional probability model from another point of view, there may be one or more decision trees,

choosing the best solution from multiple decision trees is the NP problem. Therefore, it is an approximate solution. The RF algorithm is a very representative bagging ensemble algorithm. All of the base evaluators are decision trees. Based on the random sampling of bagging samples, random selection of input features is added in RF. This model compensates for the shortcomings of decision trees by combining small decision trees instead of building large decision trees, that is, preventing possible overfitting. The AdaBoost is an abbreviation of adaptive boosting. Since the model building process from a single weak learner is one by one, the results of the previous learning will affect the building process of the latter model. By combining the output of a single weak learner into the weighted sum, the final output result is represented. The XGBoost algorithm is built by the parallel construction of regression trees through multi-threading, which can control the model during the entire training process through a series of hyperparameters to greatly improve the speed and accuracy of model training. XGBoost prevents overfitting, which is the main limitation of enhancement through regularization gradients. In other words, XGBoost create a smooth curve by reducing the differences between the data. The GBDT algorithm constructs a set of weak learners (trees) and aggregates the results of multiple decision trees as the final predictive output. Like XGBoost, it is also necessary to regularize GBDT to prevent overfitting. By using classification and regression tree (CART) based learner as weak classifier, GBDT model has better robustness and can automatically discover higher-order relationships among features. For achieving the balance between accuracy and generality via the defined inputs in ML algorithms, GridSearchCV is used to the hyperparameter tuning, which performs the search on a specified set of hyperparameters. At the same time, the *k*-fold cross-validation is used to optimize hyperparameters, which can more accurately represent the model's performance on data what has not been learned during each random sampling by randomly selecting as the testing set without replication. And in the regression task, the most important task is to minimize the error between the predicted results and the actual sample values as much as possible, therefore adjusting the parameters of the algorithm and hyperparameters minimize the generalization error.

Performance evaluation:

The *P*earson correlation coefficient (*r*), coefficient of determination (R^2), root mean square error (RMSE), mean absolute error (MAE), and mean absolute percentage error (MAPE) are often used to evaluate algorithms with the regression problems, which are calculated by Equations (1-5), respectively, As shown in the following:

$$r_{xy} = \frac{n\sum x_i y_i - \sum x_i \sum y_i}{\sqrt{n\sum x_i^2 - (\sum x_i)^2} * \sqrt{n\sum y_i^2 - (\sum y_i)^2}} \quad (1)$$

$$R^2 = \frac{\left[\sum_{i=1}^{n}(y_i' - \hat{y})(y_i' - \widehat{y'})\right]^2}{\sum_{i=1}^{n}(y_i - \hat{y})^2 \cdot \sum_{i=1}^{n}(y_i' - \widehat{y'})^2} \quad (2)$$

$$RMSE = \sqrt{\frac{\sum_{i=1}^{n}(y_i' - y_i)^2}{n}} \quad (3)$$

$$MAE = \frac{1}{m}\sum_{i=1}^{m}|(y_i - \hat{y_i})| \quad (4)$$

$$MAPE = \frac{100}{m}\sum_{i=1}^{m}\left|\frac{(y_i - \hat{y_i})}{y_i}\right|, y_i \neq 0 \quad (5)$$

Where, n represents the number of observation objects, x_i and y_i is the i-th observation of x and y in equation (1), respectively. y_i and y_i' stand for the tested and predicted values, ˆy and ˆy' are the average values of the tested and predicted values in equations (2-5), respectively. *P*earson correlation coefficient is also called the linear correlation coefficient, which is used to detect the degree of linear correlation between two continuous variables. It is clear that *r* stand for the quotient of the covariance and the standard deviation between two variables. And the higher R^2 value indicates that the predicted PCE (or J_{sc}/FF) is in good agreement with the actual experimental value. The sample deviation between predicted and measured residual values is measured by taking into account RMSE, MAE, and MAPE.

Device fabrication:

The specifications of the materials used are detailed in the manuscript Materials section and will not be repeated herein. The structure of the device is ITO/PEDOT:PSS/Active layer/PDIN/Ag. Firstly, the indium tin oxide (ITO) glass substrates with sheet resistance of 10Ω/□ are continuously cleaned in ultrasonic bathes containing special glass lotion, deionized water and ethanol for 30min in each step, and then air-dried with high-purity nitrogen. The pre-cleaned ITO substrates are treated with UV-ozone for 15min to further improve the work function of the ITO substrates. The PEDOT:PSS aqueous solution is filtered through the 0.35 mm filter and then deposited on pre-cleaned ITO substrate at 5000 rpm for 30s. Then the PEDOT:PSS film is annealed at 150℃ for 15min in air before the substrate is transferred to the glove box. For the PTQ10 system, the PTQ10 donor with NFAs are configured with a mixed solution (chloroform:1,8 diiodooctane (DIO), 99.7%:0.3%, v/v) at 1:1.4 (w/w) at a total concentration of 16.8 mg/ml, and heated and stirred at 45℃ for 2h for using. The blend solution is spin-coated on the PEDOT:PSS at 4000 rpm. For the PM6 device, the PM6 donor and NFAs are configured with mixed solution (chloroform:DIO, 99.7%:0.3%, v/v) at 1:1.2, w/w, with total concentration of 15.4 mg/ml, and heated and stirred at 50℃ for 2h for use. And the mixture is spin-coated on the PEDOT:PSS at 2600 rpm. All blend films are transferred to the heating platform and annealed at 90℃ for 5min. Then methanol solution of PDIN at a concentration of 2 mg/mL is spin-coated on the active layer at a rate of 5000 rpm for 30s to form the cathode buffer layer. Finally, about 100nm thick Ag electrode is deposited under a vacuum conditions of $1*10^{-4}$ Pa.

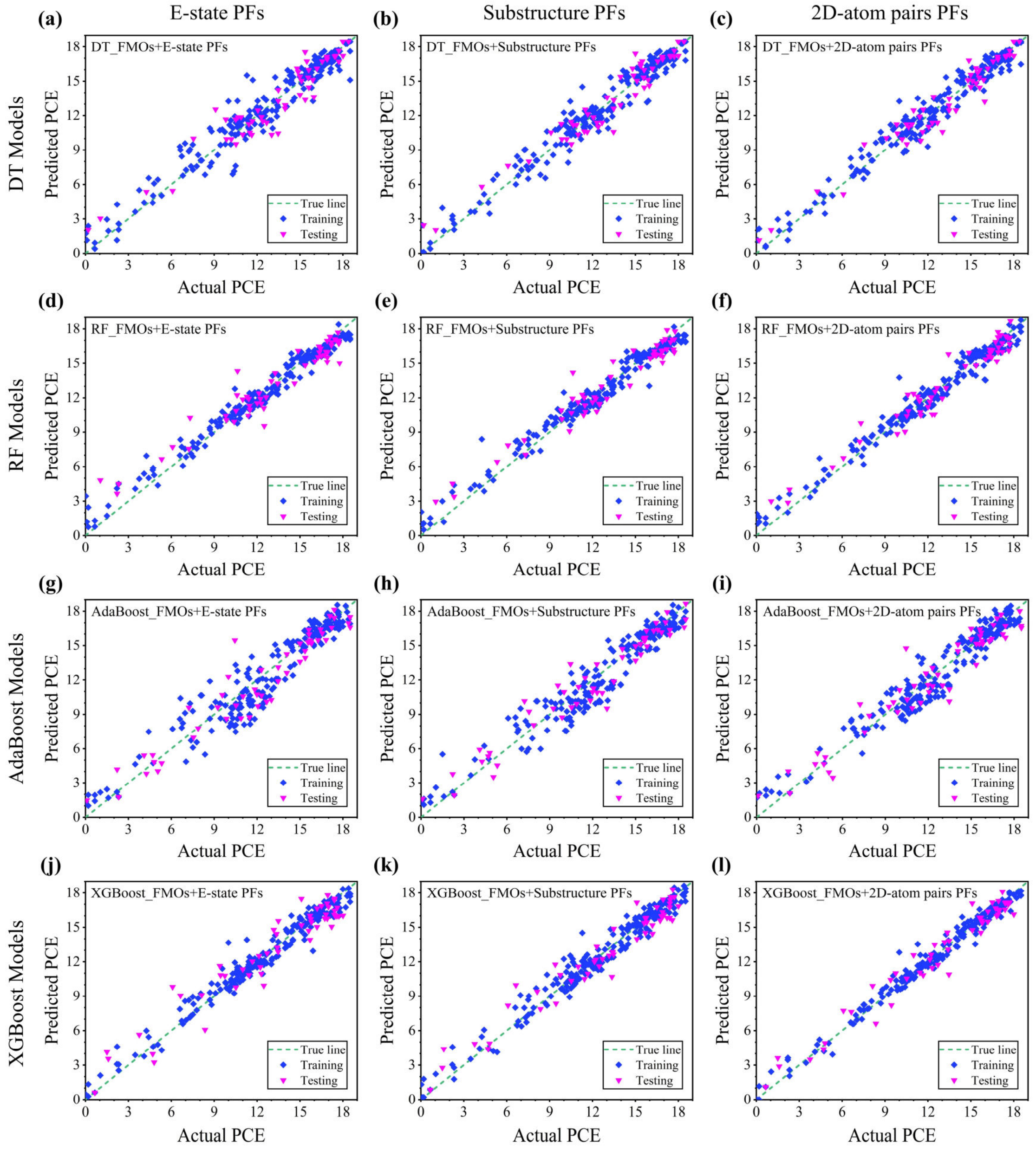

Figure S1. The fitting relationship between PCE prediction results and experimental values reported in the literature based DT, RF, AdaBoost, XGBoost, GBDT five machine learning models, respectively by using FMOs+E-state FPs, FMOs+Substructure FPs, FMOs+2D-atom pairs FPs as input features. (The blue dots represent the training data, the magenta dots indicated as the testing data, and the green dashed lines represent visual references where the predicted values are equal to the experimental values in figure.)

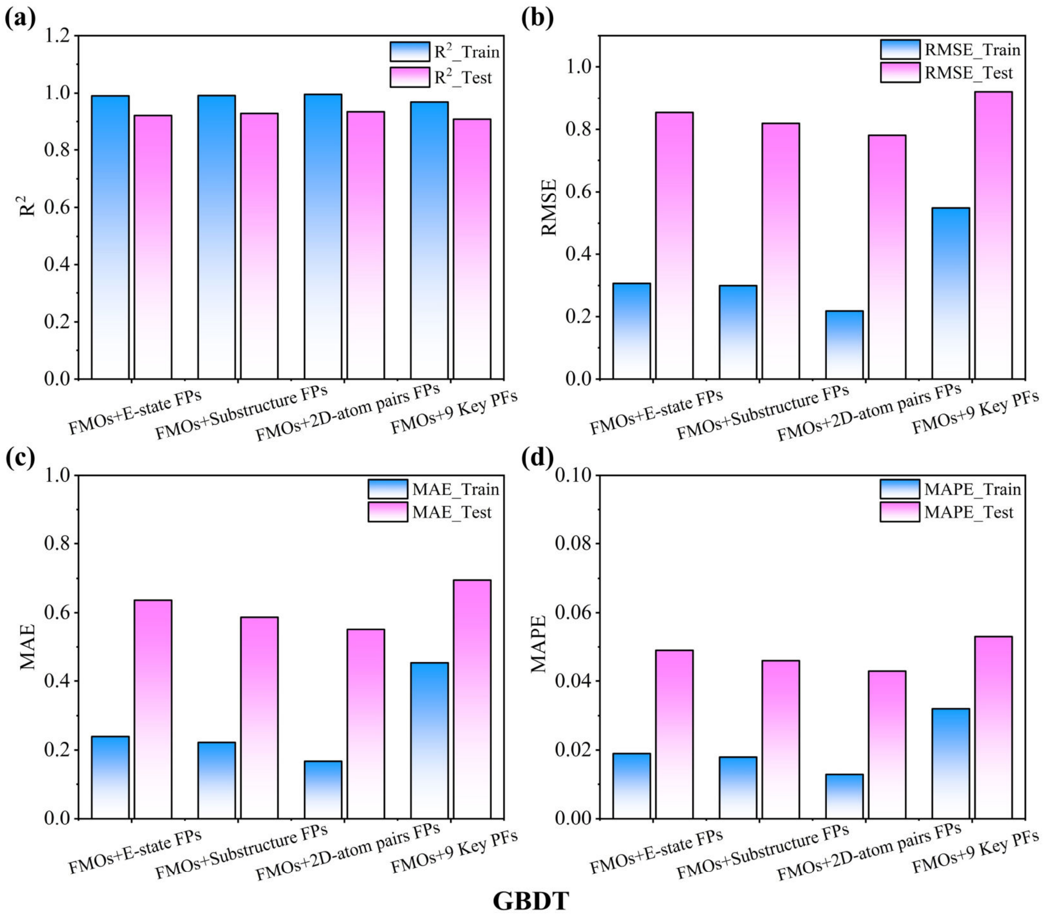

Figure S2. The performance evaluation indicators of GBDT model based on ternary data PCE prediction results on training set and testing set: (a)R^2, (b)RMSE, (c)MAE, (d)MAPE by taking FMOs+E-state FPs, FMOs+Substructure FPs, FMOs+2D-atom pairs FPs, FMOs+9 Key PFs from binary OSCs as input features, respectively.

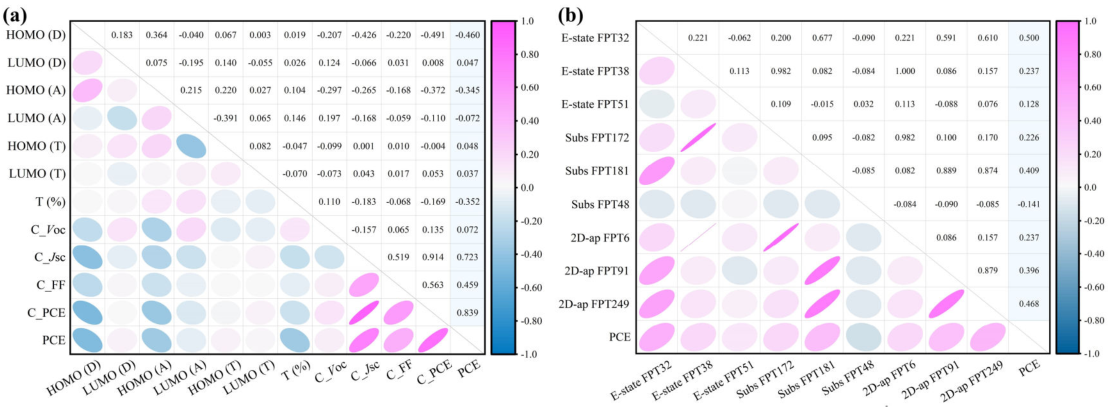

Figure S3. Correlation matrix of the PCE and (a) FMOs, T(%) and control parameter features, (b) 9 key features from the third component, in ternary PSCs.

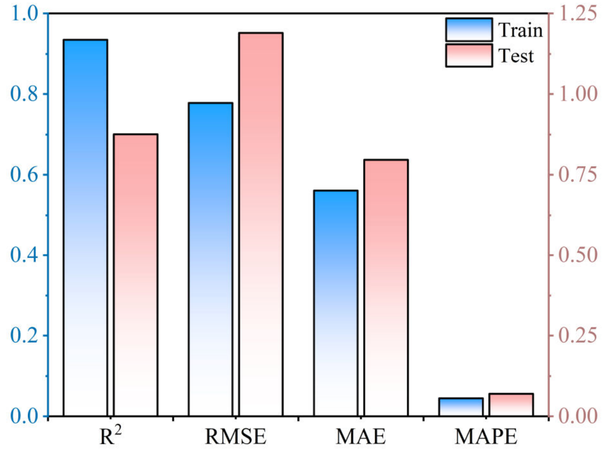

Figure S4. The performance evaluation indicators of GBDT model based on ternary data PCE prediction results on training set and testing set with FMOs+9 Key PFs from T as input feature.

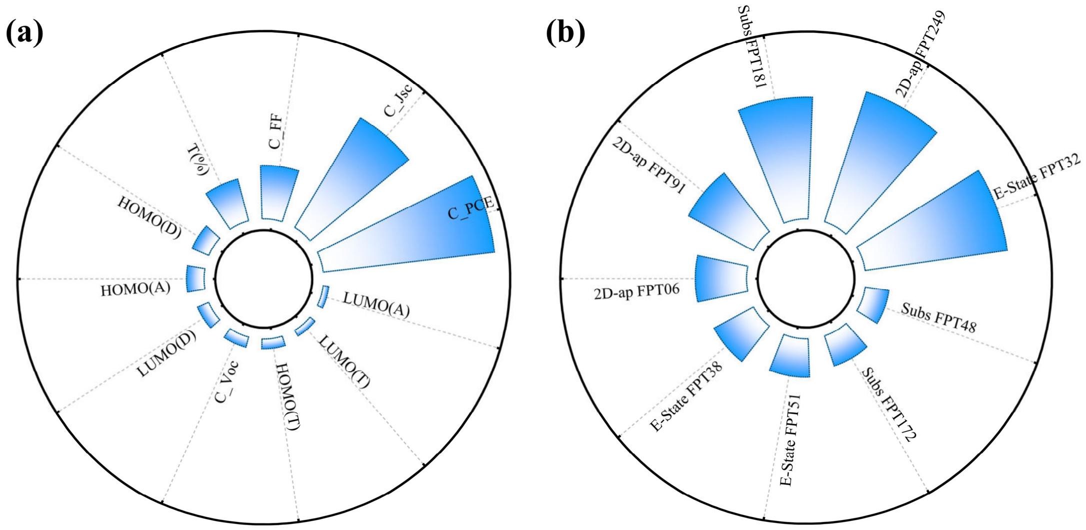

Figure S5. (a, b) Ranking the importance of the influence of key input features on PCE of ternary PSCs in GBDT model.

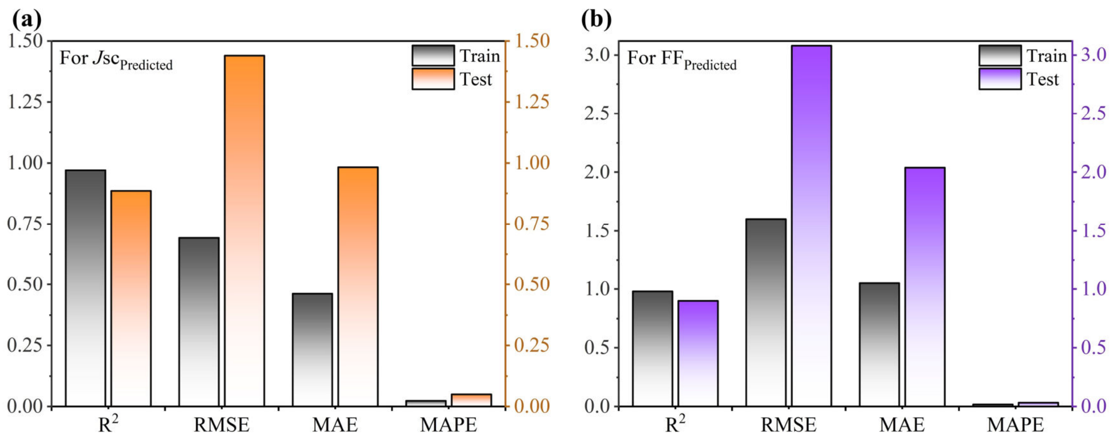

Figure S6. The performance evaluation indicators of GBDT model based on ternary data (a) J_{sc} and (b) FF prediction results on training set and testing set with FMOs+9 Key PFs from T as the input feature.

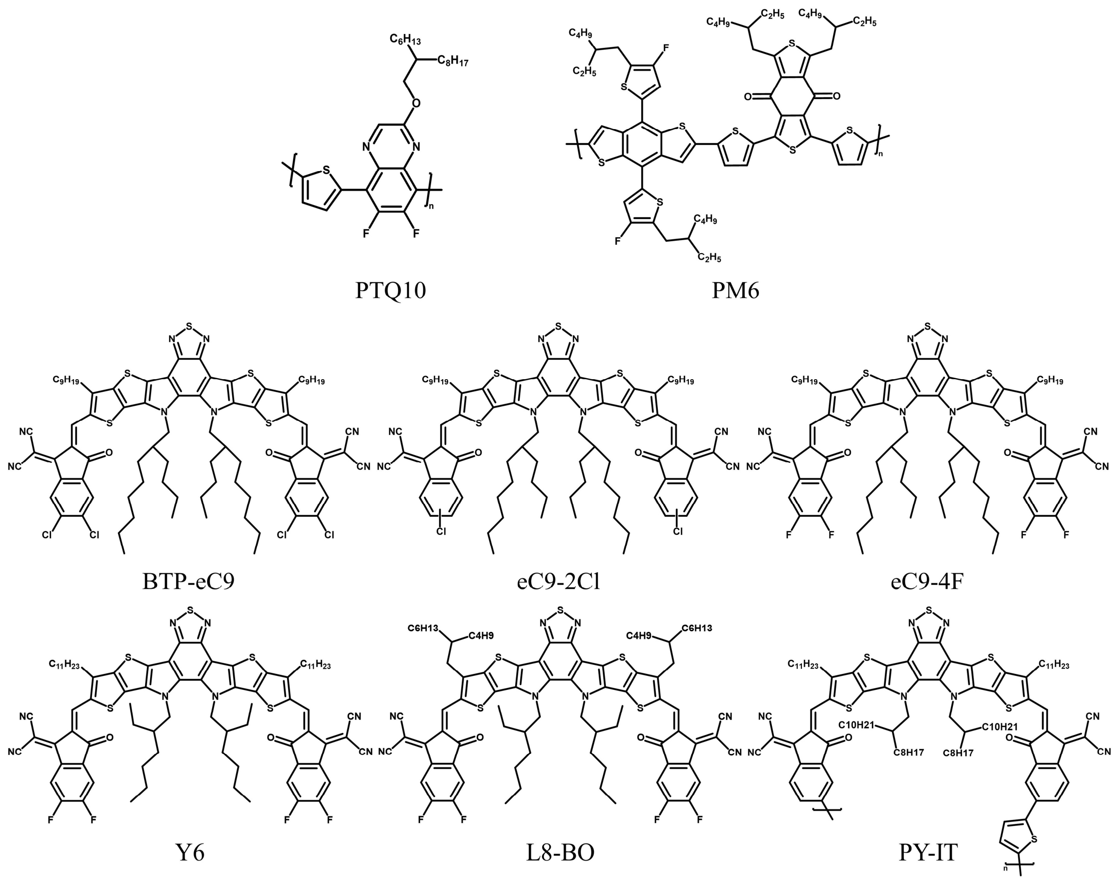

Figure S7. The chemical structure of donor, acceptor and third component materials used in this work.

Table S1. The screened 9 key molecular fingerprint descriptors from binary data.

Features	Description
E-state FPA11	dsCH ([CD2H](=*)-*)
E-state FPA35	dO ([OD1H0]=*)
E-state FPA54	sCl ([ClD1]-*)
Substructure FPA133	Isonitrile ([ClD1]-*)
Substructure FPA171	Arylfluoride ([F][c])
Substructure FPA172	Arylbromide ([Br][c])
2D-atom pairs FPA171	3_N_S (Presence of N-S at topological distance 3)
2D-atom pairs FPA190	3_S_S (Presence of S-S at topological distance 3)
2D-atom pairs FPA404	6_N_O (Presence of N-O at topological distance 6)

Table S2. The screened 9 key molecular fingerprint descriptors from ternary data.

Features	Description
E-state FPT32	aasN ([N,nD3H0](:*)(:*)-,:*)
E-state FPT38	sF ([FD1]-*)
E-state FPT51	aaS ([S,sD2H0](:*):*)
Substructure FPT48	Ketone ([#6][CX3](=[OX1])[#6])
Substructure FPT172	Arylbromide ([Br][c])
Substructure FPT181	Hetero O ([o])
2D-atom pairs FPT6	1_C_F (Presence of C-F at topological distance 1)
2D-atom pairs FPT91	2_N_N (Presence of N-N at topological distance 2)
2D-atom pairs FPT249	4_N_S (Presence of N-S at topological distance 4)

Table S3. The R^2, RMSE, MAE and MAPE are used to evaluate the GBDT model's performance for J_{sc} and FF prediction on ternary data set with FMOs+9 Key PFs from T as the input feature.

ML Models	Photovoltaic Performance	R^2 Train/Test	RMSE Train/Test	MAE Train/Test	MAPE Train/Test
GBDT	J_{sc}	0.961/0.885	0.692/1.440	0.462/0.983	0.023/0.049
	FF	0.980/0.900	1.602/3.080	1.050/2.041	0.017/0.032
